# BOLD fMRI effects of transcutaneous vagus nerve stimulation in patients with chronic tinnitus

**DOI:** 10.1371/journal.pone.0207281

**Published:** 2018-11-28

**Authors:** Natalia Yakunina, Sam Soo Kim, Eui-Cheol Nam

**Affiliations:** 1 Institute of Medical Science, Kangwon National University, School of Medicine, Chuncheon, Republic of Korea; 2 Neuroscience Research Institute, Kangwon National University Hospital, Chuncheon, Republic of Korea; 3 Department of Radiology, Kangwon National University, School of Medicine, Chuncheon, Republic of Korea; 4 Department of Otolaryngology, Kangwon National University, School of Medicine, Chuncheon, Republic of Korea; Brigham and Women's Faulkner Hospital, UNITED STATES

## Abstract

**Objective:**

Vagus nerve stimulation (VNS) is a neuromodulation method used for treatment of epilepsy and depression. Transcutaneous VNS (tVNS) has been gaining popularity as a noninvasive alternative to VNS. Previous tVNS neuroimaging studies revealed brain (de)activation patterns that involved multiple areas implicated in tinnitus generation and perception. In this study, functional magnetic resonance imaging (fMRI) was used to explore the effects of tVNS on brain activity in patients with tinnitus.

**Methods:**

Thirty-six patients with chronic tinnitus received tVNS to the inner tragus, cymba conchae, and earlobe (sham stimulation).

**Results:**

The locus coeruleus and nucleus of the solitary tract in the brainstem were activated in response to stimulation of both locations compared with the sham stimulation. The cochlear nuclei were also activated, which was not observed in healthy subjects with normal hearing. Multiple auditory and limbic structures, as well as other brain areas associated with generation and perception of tinnitus, were deactivated by tVNS, particularly the parahippocampal gyrus, which was recently speculated to cause tinnitus in hearing-impaired patients.

**Conclusions:**

tVNS *via* the inner tragus or cymba conchae suppressed neural activity in the auditory, limbic, and other tinnitus-related non-auditory areas through auditory and vagal ascending pathways in tinnitus patients. The results from this study are discussed in the context of several existing models of tinnitus. They indicate that the mechanism of action of tVNS might be involved in multiple brain areas responsible for the generation of tinnitus, tinnitus-related emotional annoyance, and their mutual reinforcement.

## Introduction

Tinnitus, a perception of phantom sound when no external sound source is present, is one of the most common and prevalent auditory disorders. The mechanism and pathophysiology of tinnitus are not fully understood, impeding the development of efficacious treatments and therapies. Although tinnitus can have many different causes, it is most commonly triggered by noise-, drug-, or age-induced damage to cochlear hair cells that causes hearing loss [1–3]. The loss of input from a specific region of the damaged cochlea leads to an imbalance between inhibitory and excitatory cortical processes, which promotes synchronous hyperactivity in the central auditory system [4–6]. Such abnormalities in neuronal behavior cause plastic reorganization of the auditory cortex, which is considered associated with the generation of tinnitus [7].

Vagus nerve stimulation (VNS) is a surgically implemented FDA-approved procedure for the treatment of epilepsy and depression [8–10]. VNS triggers the release of several neuromodulators that enhance plastic changes in the cerebral cortex [11,12]. It does so through action of a number of neuromodulators including, among others, the modulation of norepinephrine release via projections extending from the nucleus of solitary tract (NTS) to the locus coeruleus (LC), which subsequently influence the limbic, reticular, and autonomic centers of the brain [13–15]. Transcutaneous VNS (tVNS) has recently been gaining popularity as a noninvasive alternative to VNS. Noninvasiveness of tVNS makes it easier to explore brain behavior under stimulation using neuroimaging methods. Brain activation under tVNS has been shown to be similar to that under invasive VNS [16–19], and both stimulations engage the same neural pathways [20]. In a recent functional magnetic resonance imaging (fMRI) study, tVNS deactivated auditory and limbic areas, including superior temporal gyri, amygdala, hippocampus, and parahippocampal guri [19]. Limbic system has been tightly linked with tinnitus percept in Jastreboff’s classical as well as more recent models of tinnitus [21–23]. Various other non-auditory brain areas, associated with tinnitus, were deactivated by tVNS as well, such as cingulate cortex, precuneus, and frontal gyrus.

These findings raised a question of how the brain with tinnitus would react to tVNS. Although the effects of tVNS on healthy human brains has been extensively studied using various neuroimaging methods, including fMRI, the effects of tVNS on the brain of subjects with tinnitus were investigated using magnetoencephalography (MEG) in only two studies [24,25]. In the present study, the effects of tVNS on the brain of patients with chronic tinnitus were explored using fMRI, particularly the role of tVNS in activating the vagal pathway and its effect on areas implicated in tinnitus signal generation and tinnitus-related emotional distress.

## Methods

### Subjects

The present study included 36 individuals with a mean age of 51.0 ± 11.9 years (all right-handed subjects, 27 males) with chronic tinnitus lasting 3 months or longer (Table 1). The study protocol was approved by the Institutional Review Board of Kangwon National University Hospital, and all subjects provided written informed consent prior to participation. The subjects had no known ontological (other than hearing loss), neurological, or psychological disorders and were not taking any medications at the time of the experiment. Prior to the study, the stimulation procedure and the experiment protocol were explained to the subjects, who were informed that they could stop and discontinue the experiment at any time. Prior to the fMRI scanning session, subjects were familiarized with the electrical stimulation through preliminary sensory/pain threshold testing (described below), which lasted approximately 20 minutes.

**Table 1:** Characteristics of tinnitus in 36 patients.

Tinnitus characteristics	Value
Duration (months) of tinnitus	63.2 ± 59.5 (3–134)
Lateralization (bilateral/right/left)	14/11/11
Pitch (Hz)	6268 ± 4858 (125–14000)
Loudness (dB SL)	8.0 ± 4.5 (5–20)
THI score	46.4 ± 19.5 (28–71)

SL: sensation level; THI: tinnitus handicap inventory

### Hearing and tinnitus assessment

Hearing and tinnitus assessment was done in a double-walled soundproof room (ISO 6189). Pure tone air audiometry (0.25–16 kHz) and tinnitogram were performed using a MADSEN Astera audiometer running OTOsuite software (GN Otometrics, Denmark) and calibrated headphones (TDH39; GN Otometrics).

### Electrical stimulation

Based on our previous tVNS fMRI study with normal subjects, two locations were chosen for active stimulation, the inner tragus and cymba conchae [19]. Stimulation at the earlobe was performed as sham, as it is known to be relatively free of vagal innervation [26]. The electrical stimulation was applied using the custom-made stimulator [19]. The electrical stimulus was a monophasic rectangular impulse with a pulse width of 500 μs and a stimulation frequency of 25 Hz, which was shown to produce better results than low frequencies during VNS [27]. The stimulation was applied to the left ear because the efferent vagal fibers to the heart are generally located on the right side [14]. The reference electrodes for the tragus and concha were placed at the outer surface of the tragus, whereas the reference electrode for the earlobe was placed on the back side of the earlobe.

Subject preparation, intensity testing, and general experimental procedures were performed as described in our previous study [19]. Prior to each functional run, patients’ sensory and pain thresholds were tested inside the MRI scanner. The stimulation intensity for each electrode was chosen as the intensity 0.1 mA weaker than the intensity corresponding to the pain threshold. The subjects were instructed to remain still with their eyes closed. The subjects had access to an emergency button to interrupt the scanning if necessary, and they were informed that they could withdraw from the experiment at any time if they experienced discomfort. The participants were asked about their general condition throughout the imaging session.

### Data acquisition

Imaging was performed using a 3.0 T MRI scanner (Philips Achieva, Philips, Amsterdam, The Netherlands) with a 32-channel SENSE head coil (Philips). Coronal 3D T1-weighted high-resolution structural images of the whole brain were acquired for anatomical orientation using the following parameters: TR = 9.8 ms, TE = 4.8 ms, FA = 8°, slice thickness = 1.0 mm, matrix = 256 × 256 × 195, FOV = 220 × 220 mm, and voxel size = 0.94 × 0.94 mm. Additionally, T2*-weighted functional images were acquired using a gradient echo planar imaging (EPI) sequence with the following parameters: 30 oblique coronal slices, TR = 2000 ms, TE = 35 ms, FA = 90°, matrix = 80 × 80, FOV = 220 × 220 mm, and voxel size = 2.75 × 2.75 mm. The slice plane was positioned parallel to the back wall of the brainstem. Each location was stimulated in two runs with 30 s of stimulation followed by 30 s of rest; this cycle was repeated five times in a run. Each subject underwent a total of six 5-min fMRI runs, with up to 90 s of rest between runs. The order of stimulation was counterbalanced and varied from subject to subject. A total of 300 functional volumes were obtained for each stimulation location.

Throughout the entire imaging session, subjects’ heart rates were monitored using a wireless MRI-compatible pulse oximeter (Medrad Veris^TM^ 8600, Medrad, Inc., Warrendale, PA, USA) attached to the right index finger. The experiment was to terminate immediately if the subject showed bradycardia (heart rate <60 BPM) or abnormal cardiac rhythms.

### Data analysis

#### Preprocessing and general linear model

All data were preprocessed and statistically analyzed using the SPM12 software package (Wellcome Department of Cognitive Neurology, Institute of Neurology, University College London, UK) in the MATLAB 9.1 programming environment (MathWorks, Inc., Natick, MA, USA). Preprocessing included the following steps for each subject: correction for head motion, slice timing correction, co-registration to the first volume of each run, normalization to the standard Montreal Neurological Institute (MNI) T1 template, and spatial smoothing using an 8-mm isotropic Gaussian kernel.

At the individual level, the preprocessed data were fitted to a general linear model implemented in SPM12. For each run, the boxcar stimulus function was convolved with a canonical hemodynamic response function, and data were high-pass filtered using a cutoff period of 128 s. Motion parameters were added as nuisance regressors. Serial correlations in the fMRI time series were accounted for using an autoregressive AR(1) model. The blood-oxygen-level-dependent (BOLD) activity in each of the three stimulation locations was first modeled separately to obtain the stimulation–rest contrast for each electrode. Then, the stimulation data for all three locations were fitted into one model, and the tragus–earlobe and concha–earlobe contrasts were obtained to compare stimulation at the two active locations with that at the sham location. One-sample *t*-tests were performed on the resulting individual contrast maps to obtain group activation maps, and these were corrected for multiple comparisons using a cluster-significance threshold of p < 0.05 to indicate statistical significance.

#### Regions of interest (ROIs) analysis

Regions of interest (ROIs) were defined for the amygdala, hippocampus, parahippocampal gyrus (PHG) representing the limbic system, Heschl’s and superior temporal gyri representing the auditory system, LC, and NTS representing the vagal pathway [28–30]. The LC ROI was defined using an available template (http://www.eckertlab.org/LC; [31], and the NTS ROI was defined based on existing literature [32,33]. The remaining ROIs were defined using the SPM Neuromorphometrics atlas. All statistical analyses were performed using IBM SPSS software (version 22.0; IBM Corp.; Armonk, NY, USA). The percentage signal change (PSC) was calculated using the following formula: 100 × (S_stim_−S_rest_)/S_rest_, where S_stim_ was the signal intensity during the stimulus periods, and S_rest_ was the signal intensity during the resting periods. The number of voxels with t > 3.33 for activation and t < -3.33 for deactivation, the average t-score, and the PSC were calculated for each ROI for the two active locations (A and B) and compared between the locations using paired *t*-tests with Bonferroni’s correction for multiple comparisons. Stimulation intensities and sensory thresholds were compared among all locations using a within-subject analysis of variance (ANOVA) with Bonferroni’s correction for multiple comparisons.

#### Supplementary analysis: Comparison with normal subjects

We compared results for the tinnitus patients (TINN) with those obtained from normal controls (NORM) in our previous study as a supplementary analysis (Table 2) [19]. The functional volumes were matched between the studies. In our previous study, the subjects underwent 30 s of stimulation followed by a 60-s rest, repeated four times in each functional run. To match the volumes, the volumes corresponding to the last 30 s of rest in each stimulation cycle in the data from NORM and the entire last stimulation cycle data (30 s of stimulation + 30 s of rest) in TINN were removed from the analysis. As a result, the analysis was performed on two datasets of NORM and TINN that consisted of 240 functional volumes representing 30 s of stimulation followed by 30 s of rest, repeated four times in each functional run. A general linear model (GLM) was performed as described above for both datasets. A two-sample *t*-test using the SPM software was performed for the tragus and concha locations to reveal differences between NORM and TINN. ROI analysis of limbic and auditory structures was performed for the two datasets, and the results were compared using a two-sample *t*-test.

**Table 2:** Demographic and hearing characteristics of tinnitus patients and normal subjects.

	Tinnitus patients (N = 36)	Normal subjects (N = 37)
Age (years)	51.0 ± 11.9	30.9 ± 8.2
	(29–70)	(21–51)
Gender (Male)	27 (75%)	18 (48.6%)
Average hearing threshold (dB HL)		
Non-tinnitus ears	16.5 ± 22.2	9.3 ± 5.2
Tinnitus ears	21.6 ± 22.6	-
Handedness		
Right handed	36 (100%)	35 (94.6%)
Left handed	-	2 (5.4%)

HL: hearing level

## Results

The hearing thresholds were 21.6 ± 22.6 and 16.5 ± 22.2 dB HL for tinnitus and non-tinnitus ears, respectively (Table 2). All subjects had a mild degree of high-frequency hearing loss.

The sensory thresholds at the three stimulation locations were 0.1–1.4 mA, 0.2–1.4 mA, and 0.2–1.0 mA for the tragus, concha, and earlobe, respectively, with means ± standard deviations (SD) of 0.46 ± 0.25, 0.55 ± 0.35, and 0.51 ± 0.23. The stimulation intensities at all electrodes ranged from 0.1 to 1.8 mA, with means ± SD of 0.71 ± 0.43, 0.80 ± 0.47, and 0.79 ± 0.47, respectively. There were no significant differences between sensory thresholds and stimulation intensities among electrodes. No subject experienced bradycardia (heart rate <60 BPM) or abnormal cardiac behavior during the experiment. No participant withdrew or was withdrawn from the experiment.

### Comparison of stimulation versus resting states

**Fig 1:**
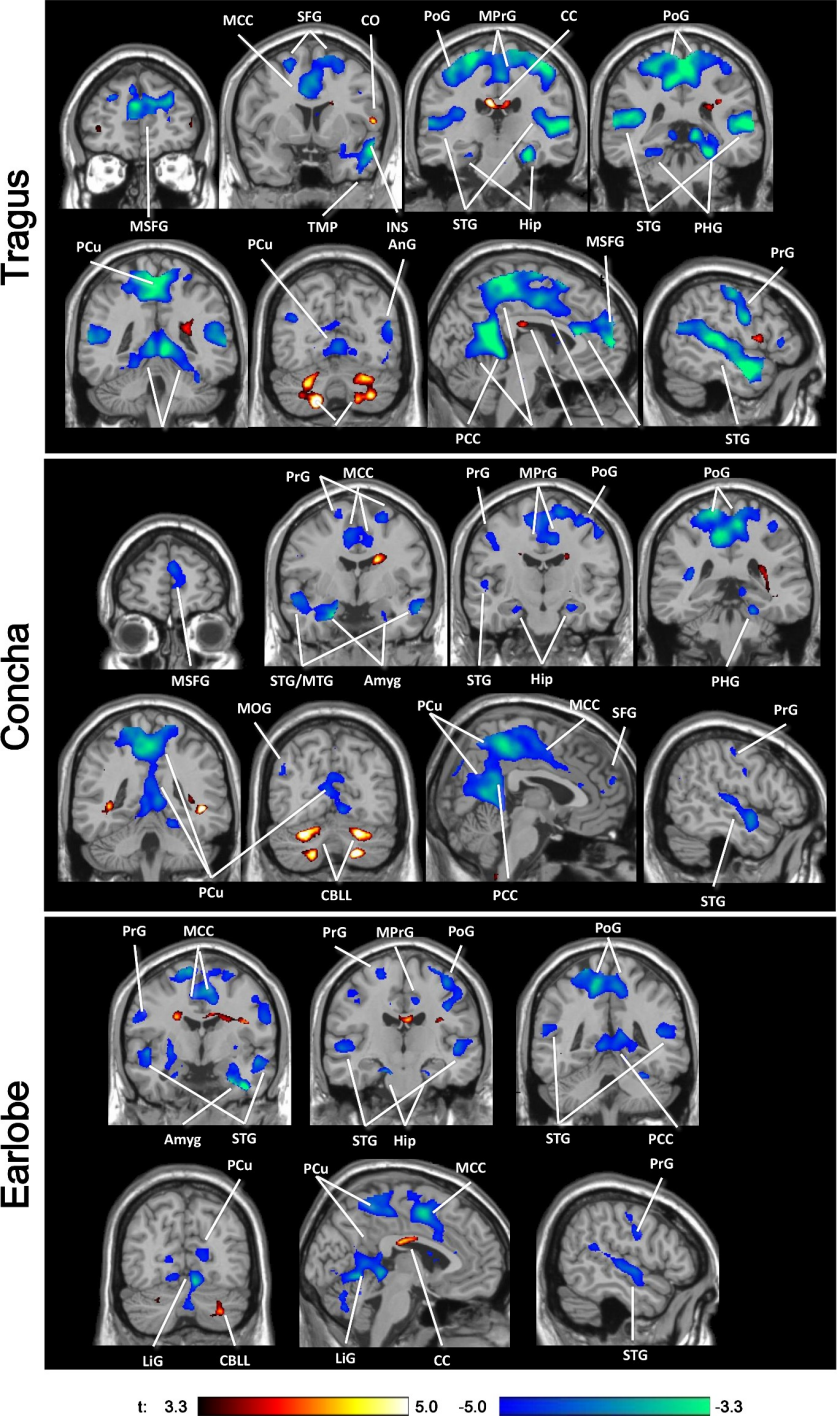
Activations (red) and deactivations (blue) induced by tVNS at the tragus, cymba conchae, and earlobe (p < 0.05, cluster corrected for multiple comparisons). tVNS: transcutaneous vagus nerve stimulation; ACC/MCC/PCC: anterior/middle/posterior cingulate cortex; Amyg: amygdala; AnG: angular gyrus; CC: corpus callosum; CBLL: cerebellum; FuG: fusiform gyrus; Hip: hippocampus; LiG: lingual gyrus; MOG: middle orbital gyrus; MTG/STG: middle/superior temporal gyrus; PCu: precuneus; PoG/PrG: postcentral/precentral gyrus; SFG: superior frontal gyrus; TMP: temporal pole.

The group analysis results of the three experimental locations relative to baseline are presented in Table 3 and Fig 1. Stimulation at the tragus and concha caused bilateral suprathreshold activation in the cerebellum and right caudate nucleus. Stimulation at the tragus and earlobe produced bilateral activation in the corpus callosum. The earlobe electrode additionally produced activation in the right cerebellar hemisphere.

**Table 3:** Activated and deactivated regions revealed by general linear model analysis.

				Contrast	
	tragus	concha	earlobe	tragus– earlobe	concha– earlobe
Heschl's gyrus	↓b	↓l			
Planum polare	↓b	↓l			
Planum temporale	↓b	↓l			
Temporal gyrus middle	↓r		↓r		r
Temporal gyrus superior	↓b	↓b	↓b		
Amygdala		↓b	↓r		
Hippocampus	↓r	↓b	↓b		
Parahippocampal gyrus	↓b	↓b			
Cerebellar hemisphere	↑b	↑b	↑r	b	b
Entorhinal area	↓r	↓l			
Fusiform gyrus	↓r	↓r			
Frontal gyrus medial	↓b	↓b			
Frontal gyrus middle	↓r	↓r		l	
Frontal gyrus superior	↓b	↓b			
Cingulate gyrus anterior	↓b				
Cingulate gyrus middle	↓b	↓b	↓b		
Cingulate gyrus posterior	↓b	↓b	↓b		
Precuneus	↓b	↓b	↓b		
Angular gyrus	↓b				
Postcentral gyrus	↓b	↓b	↓b		
Precentral gyrus	↓b	↓b	↓b	r	r
Supplementary motor cortex	↓b	↓b	↓b		
Supramarginal gyrus					l
Caudate	↑r	↑r			
Putamen			↓r		
Lingual gyrus	↓b		↓b		
Corpus callosum	↑b		↑b		

Results are presented at *p* < 0.05, FDR, cluster-wise corrected for multiple comparisons.

↑: activation; ↓: deactivation; r: right; l: left, b: bilateral.

Stimulation at all three electrode sites produced deactivation in the auditory and auditory-associated cortices in the superior (all three locations bilaterally) and middle temporal gyri (tragus and earlobe on the right side), Heschl’s gyrus, planum polare, and planum temporale (tragus bilaterally, earlobe on the left side).

In the limbic system, deactivation was observed in the posterior cingulate gyrus and hippocampus for all electrode locations, in the amygdala for electrodes at the concha and earlobe, and in the PHG for the tragus and concha sites. Deactivation was also observed in several frontal and occipital regions including the precuneus, occipital, lingual, fusiform, postcentral, precentral, middle/anterior cingulate, and medial/middle/superior frontal gyri (Table 3, Fig 1).

### Comparison of active location stimulations (A and B) with the sham stimulation (C)

**Fig 2:**
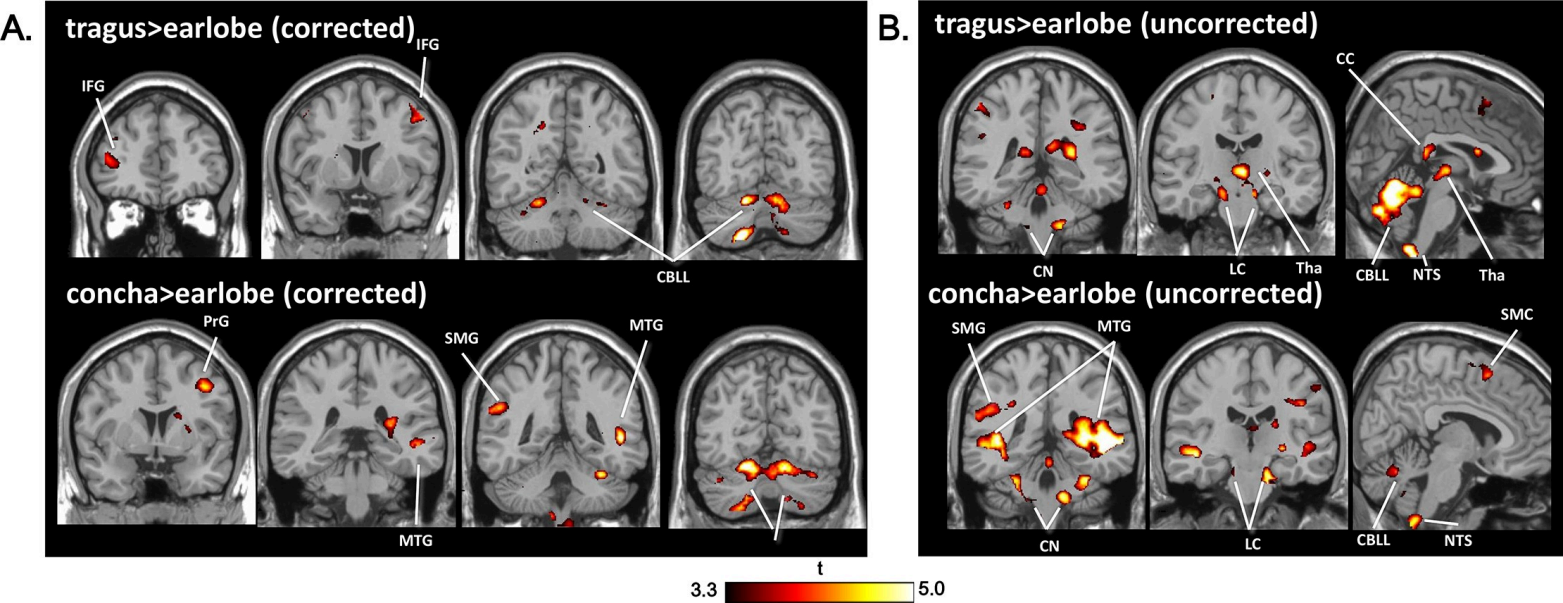
**Spatial maps showing differences between the active stimulation locations and the sham stimulation location, corrected (A) and uncorrected (B) for multiple comparisons.** CBLL: cerebellum, CC: corpus callosum, CN: cochlear nucleus, IFG: inferior frontal gyrus, LC: locus coeruleus, MTG: middle temporal gyrus, NTS: nucleus of solitary tract, PrG: precentral gyrus, SMC: supplementary motor cortex, SMG: supramarginal gyrus, Tha: thalamus.

Compared with the sham, stimulation at the tragus and concha produced increased activity in the bilateral cerebellum and right precentral gyrus (Fig 2A). Additionally, the tragus–earlobe contrast showed increased activity in the left middle frontal gyrus, and the concha–earlobe resulted in increased activity in the left supramarginal gyrus and right middle temporal gyrus. When examining the uncorrected maps of comparisons with the sham stimulation (p < 0.001), activations of the NTS, LC, and cochlear nucleus (CN), as well as several additional cortical activations were observed on both difference maps (Fig 2B).

### ROI analysis

**Fig 3:**
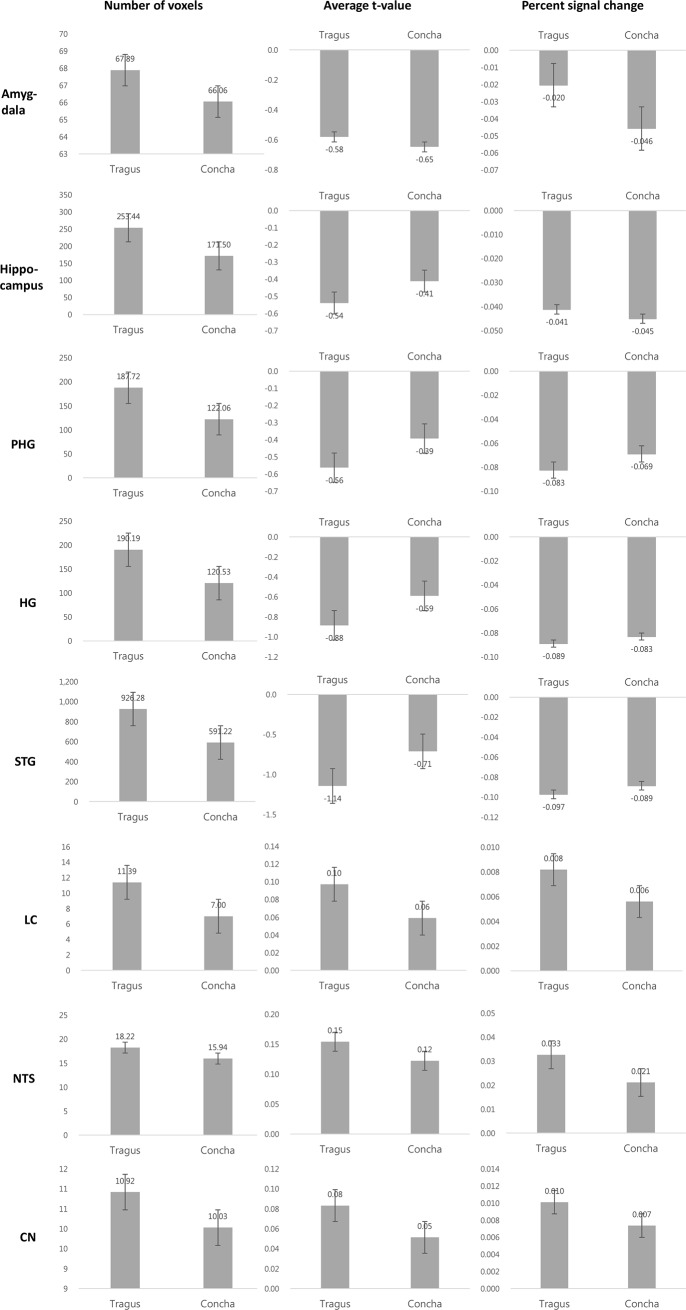
Results of ROIs analysis in tinnitus patients: number of voxels, average t-value, and PSC in the auditory, limbic, and vagal brainstem structures for each electrode location. Error bars represent standard error. ROIs; regions of interest; PSC: percent signal change; CN: cochlear nucleus; HG: Heschl’s gyrus; LC: locus coeruleus; NTS: nucleus of solitary tract; PHG: parahippocampal gyrus; STG: superior temporal gyrus. No significant differences were found among the locations.

Similar to the GLM results, the auditory and limbic areas showed negative t-scores and PSC for both the tragus and concha locations (Fig 3). LC, NTS, and CN, although only visible on the difference maps, showed positive t-scores and PCS. Differences between the locations were not observed for any ROI.

### Supplementary analysis: Comparison with normal subjects

**Fig 4:**
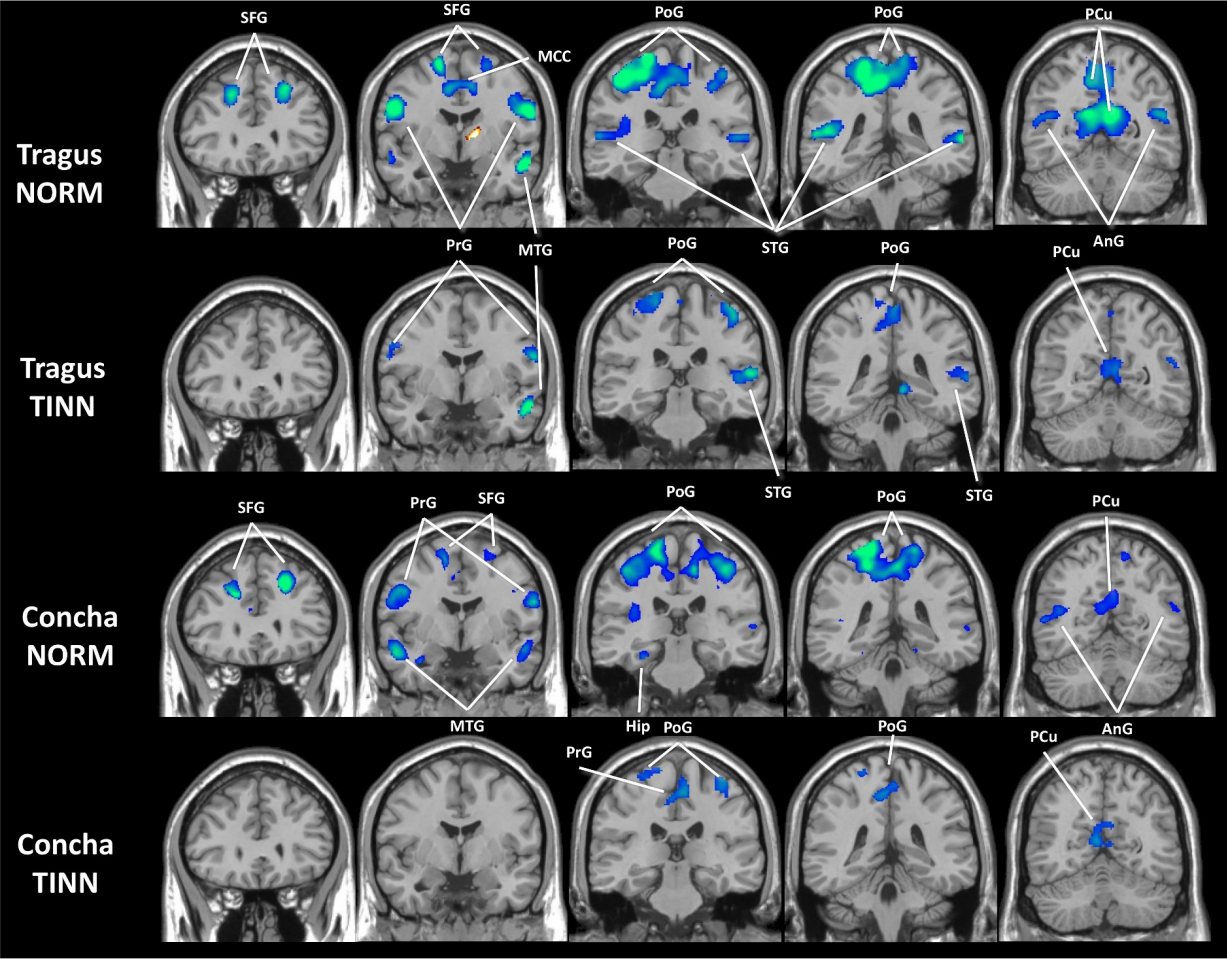
Activations (red) and deactivations (blue) induced by tVNS at the tragus and cymba conchae for NORM and TINN after matching the number of functional volumes (p < 0.05, cluster corrected for multiple comparisons). NORM: normal subjects; TINN: tinnitus patients; AnG: angular gyrus; CC: corpus callosum; Hip: hippocampus; MCC/PCC: middle/posterior cingulate cortex; MTG/STG: middle/superior temporal gyrus; PCu: precuneus; PoG/PrG: postcentral/precentral gyrus; SFG: superior frontal gyrus.

**Fig 5:**
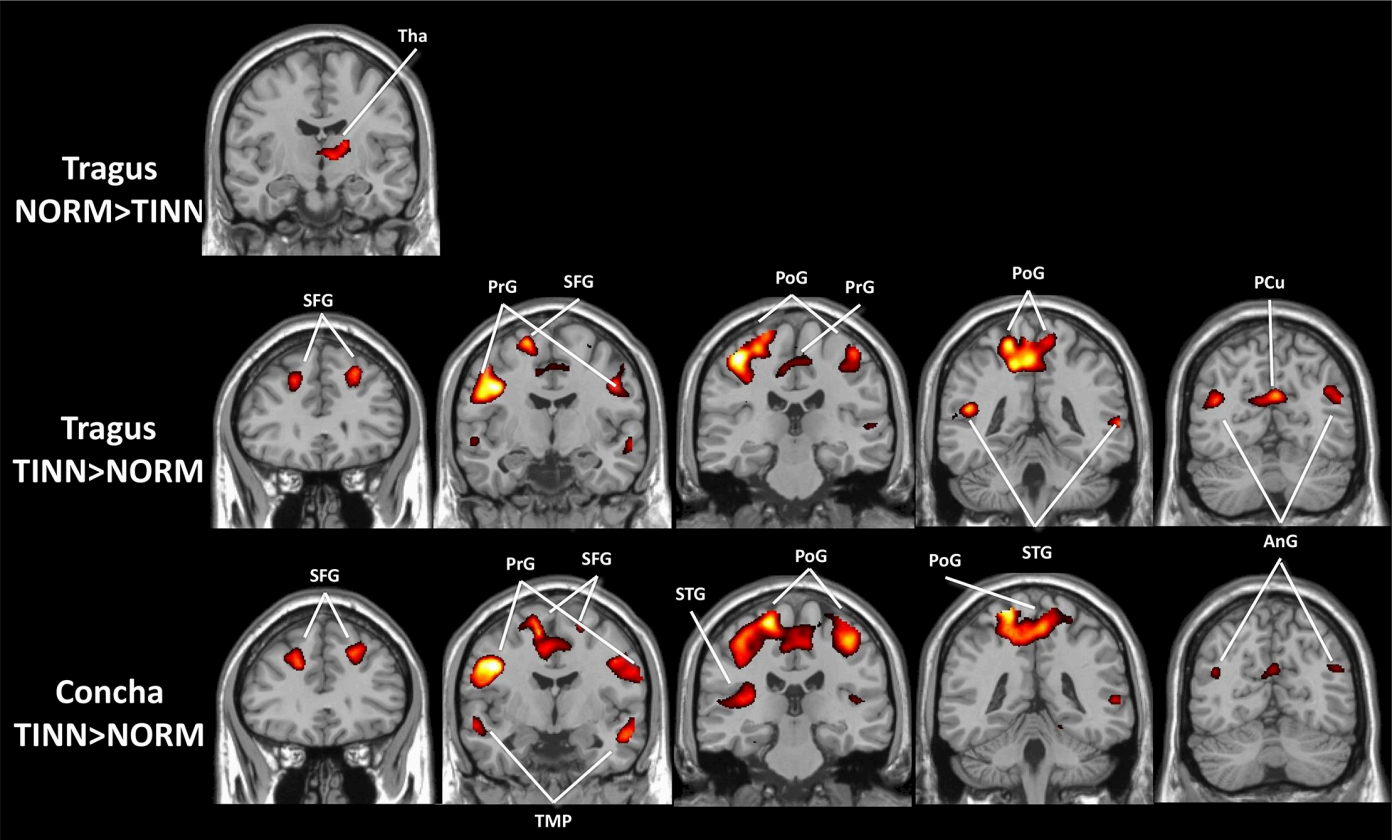
Spatial maps of the differences between the NORM and TINN datasets (p < 0.05, cluster corrected for multiple comparisons). NORM: normal subjects; TINN: tinnitus patients; AnG: angular gyrus; PCu: precuneus; PoG/PrG: postcentral/precentral gyrus; SFG: superior frontal gyrus; STG: superior temporal gyrus; TMP: temporal lobe.

The GLM results of TINN and NORM groups had similar (de)activation patterns, although NORM deactivation appeared greater (Fig 4). The only result showing NORM > TINN was for the right thalamus in response to tragus stimulation (Fig 5). On the TINN > NORM difference maps, both locations produced similar patterns, including the superior frontal, precentral, postcentral, superior temporal and angular gyri, and precuneus. Notably, this activity on TINN > NORM maps indicated weaker deactivation rather than stronger activation in TINN, as all areas examined were originally deactivated in both datasets.

**Fig 6:**
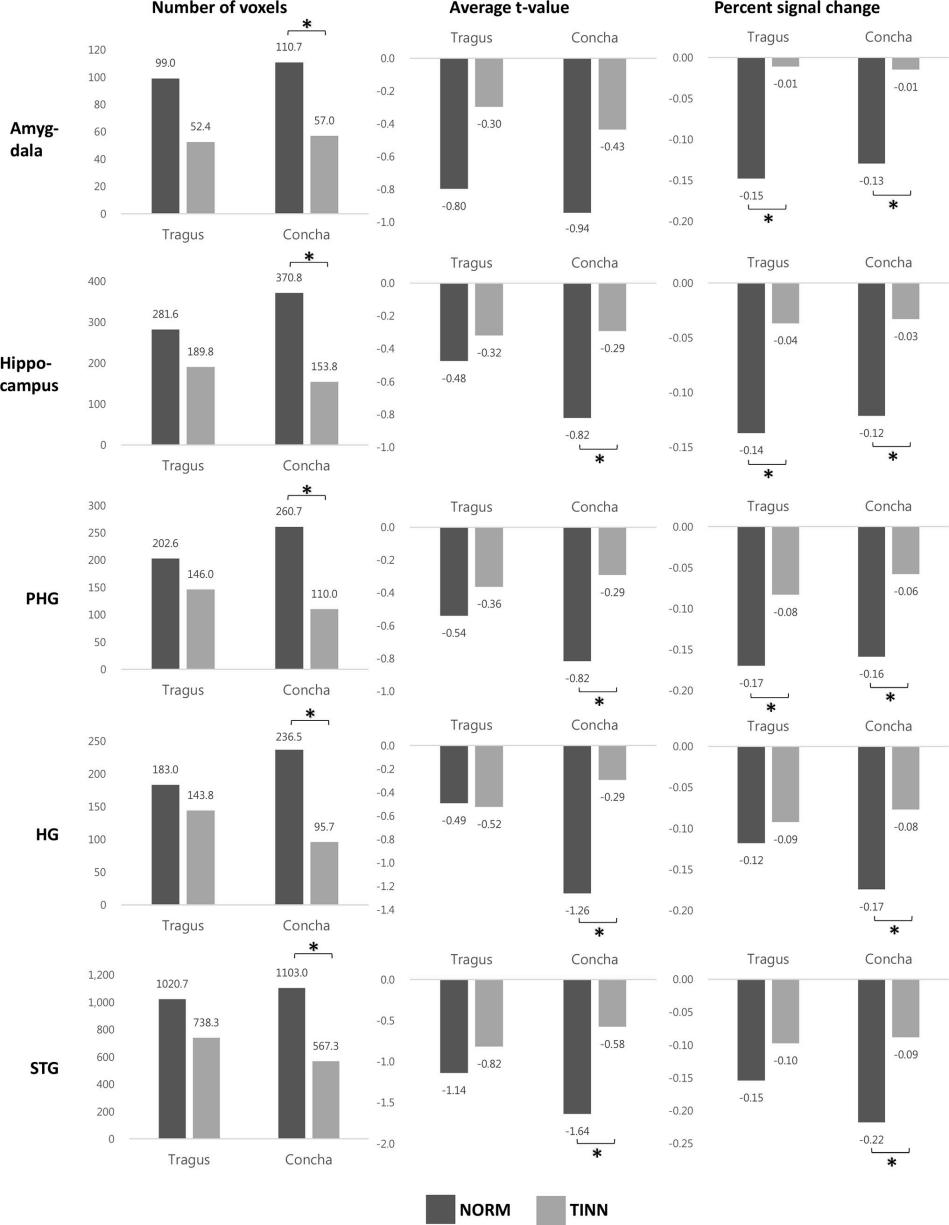
Results from the ROIs analysis in NORM and TINN. The number of voxels, average t-value, and PSC of each ROI for NORM and TINN after matching the number of functional volumes. ROIs: regions of interest; NORM: normal subjects; TINN: tinnitus patients; PSC: percent signal change; HG: Heschl’s gyrus; PHG: parahippocampal gyrus; STG: superior temporal gyrus. *: p < 0.05 (paired *t*-test, Bonferroni-corrected for multiple comparisons).

Further comparison of ROI analysis results between the two datasets showed that several deactivated voxels, average t-score, and PSC values were significantly greater in NORM than in TINN for all ROIs for the concha location, and PSC was greater in the limbic areas with stimulation of the tragus location (Fig 6).

## Discussion

In one of the two existing MEG tVNS studies, during the tVNS-on state, the amplitude of the auditory-evoked N1m response was decreased in tinnitus patients [24]. In the other study, tVNS modulated tinnitus-related beta- and gamma-band activities [25]. We utilized fMRI because it provides better spatial representation than MEG does, and the effects of the tVNS on multiple brain networks can be directly observed. To the best of our knowledge, no other fMRI study of the effects of tVNS on the brain of patients with tinnitus has been performed to date.

A recent technique for tinnitus treatment uses (t)VNS paired with acoustic stimuli for reversing tinnitus-related brain plasticity. VNS paired with tones outside the tinnitus frequency was first demonstrated to reverse tinnitus-related plastic changes in a study using rat brains [34]. This study was followed by several human studies in which tVNS paired with acoustic stimuli was shown effective in reducing tinnitus symptoms [24,35–38]. The VNS+sound therapy is based on the theory that the auditory cortex undergoes tonotopic plastic reorganization following the loss of input from the damaged cochlea [39,40]. The acoustic stimuli stripped of the tinnitus frequency supposedly increase the number of non-tinnitus frequency neurons and decrease the cortical overrepresentation of the tinnitus frequency *via* lateral inhibition [41,42]. The VNS component facilitates the plastic changes through the combined action of several neuromodulators such as acetylcholine, norepinephrine, serotonin, and GABA [12,43–45]. In the present study, tVNS successfully activated LC and NTS, as can be seen on the maps of difference with sham (Figs 2B and 3), which means that vagal pathway was activated. In addition, tVNS deactivated the auditory system (including superior temporal gyrus, Heschl’s gyrus, planum porale, and planum temporale; Table 3, Figs 1 and 3), which is in agreement with the results from the MEG study [24]. Therefore, the VNS in combined therapy hypothetically may cause general deactivation of the auditory cortex, further enhancing the sound-stimuli-induced selective suppression of tinnitus-related areas in the auditory cortex.

Based on our results, tVNS also induced deactivation of the limbic system. In Jastreboff’s classical neurophysiology model of tinnitus, the abnormally strengthened connections among the auditory, limbic, and attention systems were responsible for the generation of tinnitus and the related emotional burden [23]. The vicious cycle between those systems intensifies the tinnitus neural signal, and tinnitus becomes chronic, more annoying, and intractable. Recently, a novel tinnitus mechanism was proposed that assumes that tinnitus can be generated without the maladaptive plastic changes in the auditory cortex [46]. When hearing loss occurs, the brain attempts to fill in the missing information rather than adjust to the missing input. In cases of severe hearing loss, when the auditory system cannot further compensate for the missing auditory input, auditory memory-related areas become involved to fill in the missing information. The PHG was suggested to constitute the main node of entry for auditory information to the medial temporal lobe memory system, where salient information is encoded into long-term memory [47]. The PHG has been consistently identified in neuroimaging studies of tinnitus [48–52]. Results from a recent study confirmed the new tinnitus model, showing evidence of two tinnitus mechanisms: auditory-cortex-related tinnitus associated with little or no hearing loss, and parahippocampus-related tinnitus associated with more severe hearing loss [53]. In our study, the PHG was deactivated following the stimulation of two vagal locations (Figs 1 and 3, Table 3). tVNS also deactivated the amygdala and hippocampus, limbic areas involved in memory mechanisms along with the PHG, contributing to the persistent perception of tinnitus [21,54]. These limbic areas constitute a limbic “distress network” closely associated with phantom perception and play a critical role in Jastreboff’s model of tinnitus [21–23].

tVNS also suppressed other areas consistently implicated in the perception of tinnitus (Table 3). The anterior/posterior cingulate cortex, precuneus, and frontal cortex are considered involved in the perceptual network that raises awareness of tinnitus [21,55–57]. In addition, the medial and middle frontal gyri showed increased response in the Stroop task in tinnitus patients, suggesting a deficit in top-down cognitive control and lack of inhibitory modulation that contributes to maintaining tinnitus by hindering habituation mechanisms [56]. Aberrant neuronal activity in the precentral gyrus and supplementary motor area of tinnitus patients [48,58,59] was hypothesized as responsible for part of the conscious perception of the phantom sound [60,61]. Numerous non-auditory areas, including those mentioned above, are involved in the perception of tinnitus and should be considered targets for tinnitus treatment.

In several studies, the importance of serotonin or GABA depletion in the development of tinnitus and efficacy of pharmacological tinnitus treatment based on the enhancement of the two neurotransmitter actions was reported [62–67]. VNS evokes the secretion of norepinephrine in the LC, which facilitates serotonin secretion in the raphe nuclei [12,68]. (t)VNS has also been shown to modulate GABA receptors and increase GABA concentration [45,69,70]. Therefore, tVNS may also assist in serotonin- and GABA-mediated tinnitus suppression.

Notably, tVNS activated the CN, as shown on the difference maps for both the tragus and concha locations (Fig 2B). VNS-induced activation of the CN, along with the NTS, LC, and raphe nucleus, was reported in an animal study using immunolabeling in rats [71]. However, CN activation has not been reported in human tVNS studies. The CN is the first auditory center where integration of auditory and somatosensory information begins through convergence of afferent projections from the auditory nerve and the trigeminal/dorsal column and nuclei [72]. In subjects with hearing loss, cochlear damage and the subsequent loss of auditory input promote synaptic reorganization, which results in upregulated somatosensory inputs *via* redistribution of vesicular glutamate transporters in the CN [73,74]. Therefore, the increased influence of somatosensory input (from the tVNS) in tinnitus patients with hearing loss may enhance the tVNS-induced CN activation, which would explain the CN activation observed only in tinnitus patients and not in healthy subjects with normal hearing.

The comparison with normal subjects was limited because the normal and tinnitus groups were not matched for age, gender, or hearing function, and their stimulation protocols were slightly different. That was the reason we performed it as a supplementary analysis. If there had been a difference in the results between the two groups, it would not be appropriate to attribute it solely to tinnitus because of other factors involved. However, in spite of the difference in initial characteristics between the two groups, we found the patterns of (de)activation in tinnitus patients were similar, albeit weaker, to those in normal subjects (Figs 4–6). Therefore, it is reasonable to assume that tVNS in tinnitus patients results in the suppression of auditory and limbic structures and activation of vagus-related brainstem nuclei similarly to its action in the normal subjects. A weaker response in tinnitus patients could be attributed to two factors: older age of the tinnitus patients and difference in resting periods during tVNS stimulation between the two groups. Normal subjects had a minute of rest after 30 s of stimulation, while the tinnitus group only rested for 30 s, which might have allowed for a longer time for MRI signal relaxation during the resting period for normal subjects and possibly produced greater signal contrast between stimulation and rest. Regarding age, tVNS is a relatively young technique and age as a factor influencing the response to tVNS has not been explored yet. However, individuals of older age have shown reduced response in transcutaneous electrical stimulation (TENS) [75,76] and transcranial direct current stimulation (tDCS) [77,78].

Previous tVNS fMRI studies demonstrated activations in various brain regions in respose to tVNS, such as the insula, amygdala, hippocampus, thalamus, cerebellum, cingulate gyrus, postcentral gyrus, etc., although the activated and deactivated areas were not entirely consistent among these studies [16–19]. In contrast, not much activation was found in our study following tVNS stimulation. The stimulation protocols, parameters and analysis methods differed substantially among previous and our study, which might have contributed to the differences in results. In addition, the older age of the tinnitus group should be considered as a factor possibly reducing the strength of the response to tVNS, as was discussed earlier.

In conclusion, tVNS of the inner tragus and cymba conchae in patients with tinnitus successfully suppressed the auditory, limbic, and other brain areas implicated in the mechanisms involved in the generation/perception of tinnitus *via* auditory and vagal ascending pathways. Therefore, it appears that tVNS can potentially assist in reducing the generation and perception of tinnitus symptoms. Our study encourages further controlled clinical studies focusing on applicability and effectiveness of tVNS with and without paired sounds for the treatment of tinnitus.
